# A phase I/II, non-randomized, feasibility/safety and efficacy study of the combination of everolimus, cetuximab and capecitabine in patients with advanced pancreatic cancer

**DOI:** 10.1007/s10637-012-9802-1

**Published:** 2012-02-25

**Authors:** Sil Kordes, Dick J. Richel, Heinz-Josef Klümpen, Mariëtte J. Weterman, Arnoldus J. W. M. Stevens, Johanna W. Wilmink

**Affiliations:** Department of Medical Oncology, Academic Medical Center Amsterdam, Meibergdreef 9, 1105 AZ Amsterdam, The Netherlands

**Keywords:** Pancreatic cancer, Everolimus, Cetuximab, Capecitabine, Phase I, Phase II

## Abstract

*Background* Improvements in knowledge of molecular mechanisms in cancer are the basis for new studies combining chemotherapy with targeted drugs. Inhibition of the epidermal growth factor receptor (EGFR) by erlotinib or cetuximab has limited or no activity, respectively, in pancreatic cancer. The crosstalk between EGFR and mammalian target of rapamycin (mTOR) pathways is a potential mechanism of resistance; therefore we conducted a study to explore safety and efficacy of multiple pathway inhibition by cetuximab and everolimus in combination with capecitabine. *Methods* Safety and efficacy of fixed standard dose cetuximab in combination with various dose levels of everolimus (5–10 mg/day) and capecitabine (600–800 mg/m^2^ bid, 2 weeks every 3 weeks) were investigated in a phase I/II study in patients with advanced pancreatic cancer. The primary endpoint was objective response. *Results* Sixteen patients were treated in the phase I part at two dose levels. Mucositis, rash and hand-foot syndrome were dose-limiting toxicities. Dose level 1 (everolimus 5 mg/day, capecitabine 600 mg/m^2^ bid for 2 weeks every 3 weeks and cetuximab 250 mg/m^2^ weekly) was considered the maximum tolerated dose (MTD). Of 31 patients in the phase II part, partial response was documented in two patients (6.5%) and five (16.1%) had stable disease. Median overall survival was 5.0 months (CI 3.1–6.8). *Conclusion* The schedule of capecitabine, everolimus and cetuximab resulted in considerable epidermal and mucosal toxicities and prevented escalation to optimal dose levels. Because of toxicity and low efficacy this treatment combination cannot be recommended for treatment in pancreatic cancer patients.

## Introduction

Pancreatic cancer patients have one of the worst prognoses among all cancer types with an overall survival rate of less than 5% [1]. Since the publication of Burris in 1997 gemcitabine is still considered as the standard treatment for most patients with pancreatic cancer [2]. Several randomized clinical trials comparing gemcitabine in combination with other chemotherapeutic drugs have not resulted in survival improvement [3].

Moving away from the paradigm that combination therapy must be gemcitabine based, a strategy that has invariably failed, Conroy et al. recently demonstrated in a randomized clinical trial a significant survival advantage with the gemcitabine free combination chemotherapy regimen FOLFIRINOX as compared with gemcitabine [4]. Although patients were highly selected and toxicity was considerable, this trial opens the way to new treatment strategies in advanced pancreatic cancer.

Significant improvements in our knowledge of the molecular mechanisms involved in cancer development and progression, and the availability of drugs interfering with aberrant activity in various signaling pathways, have subsequently resulted in numerous clinical trials combining conventional chemotherapy with various targeted drugs. The EGFR/MAPK and PI3K/Akt/mTOR pathways are often dysregulated and considerable evidence supports the important role of these pathways in the biology of pancreatic cancer [5, 6]. Several targets in these pathways are potential candidates to achieve inhibition of aberrant signaling. Erlotinib, a tyrosine kinase EGFR inhibitor, was one of the first FDA approved tyrosine kinase inhibitors. In a randomized clinical trial in pancreatic cancer patients, erlotinib in combination with gemcitabine induced a statistically significant improvement in survival, although the two weeks survival benefit was considered clinically not meaningful [7]. Targeting EGFR with the monoclonal antibody cetuximab in combination with gemcitabine failed to demonstrate a survival advantage[8]. mTOR is an important signaling molecule in the PI3K pathway and inhibition of mTOR could inhibit tumor growth in pancreatic cancer xenograft models [9]. However, in a clinical study no benefit was demonstrated using the mTOR inhibitor everolimus as a single agent in second line [10]. Possible explanations for the relative insensitivity to drugs targeting only one aberrant molecule is the heterogeneous molecular pathogenesis of pancreatic cancer leading to deviant activation of multiple signaling pathways and the intensive crosstalk between these pathways [11, 12]. Although some tumors with specific crucial mutations are sensitive to mono-targeted therapies, such as gastrointestinal stromal tumor and imatinib, for most cancer types including pancreatic cancer this is not the case [13, 14]. Exploration of drug combinations targeting multiple pathways is therefore an interesting strategy to overcome drug resistance. Rational targets for this combined approach in pancreatic cancer are EGFR and mTOR, leading to synergistic anticancer activity as has been demonstrated in pre-clinical models [15–20].

Therefore we explored in the present study the feasibility and efficacy of a triple drug combination consisting of cetuximab, everolimus and capecitabine in patients with advanced pancreatic cancer. In an earlier phase I study we demonstrated that everolimus (10 mg daily) in combination with capecitabine (1000 mg/m^2^ BID) was a safe and tolerable regimen [21]. In the present study gemcitabine was replaced by capecitabine because gemcitabine in combination with everolimus induced severe bone-marrow toxicity already at the gemcitabine dose level of 600 mg/m [22]. The failure of gemcitabine based combination regimens was also taken in to account. The monoclonal antibody cetuximab instead of erlotinib was chosen because of potential pharmacokinetic interactions between erlotinib and mTOR inhibitors (at the level of cytochrome P (CYP) metabolizing enzymes) [23, 24].

## Patients and methods

### Study design and statistics

This multicenter open-label phase I/II trial consisted of two parts: the phase I part was traditionally designed with interpatient dose escalation in cohorts of three to six patients with the primary end point of protocol-defined dose limiting toxicity (DLT) and Maximum Tolerated Dose (MTD) [25]. The phase II part was designed to determinate the efficacy and feasibility of the combination of everolimus, capecitabine and cetuximab. Primary endpoint of this part of the study was response rate. Patients were defined as responders when a complete response (CR) or partial response (PR) by response evaluation criteria in solid tumors (RECIST) 1.0 was seen. Secondary endpoints were time to treatment failure (TTF), overall survival (OS), one-year survival rate and the toxicity profile according to NCI–CTC v3.0. TTF and OS were calculated by the Kaplan-Meier method, measured from the date of treatment initiation to the date of documented progression and death of any cause, respectively. All analyses were conducted on an intention-to-treat basis and were performed using SPSS version 18.0.2.

The phase II part was designed in two stages (Simon two-stage optimal design) with an early stopping rule for *efficacy*: if no objective responses were to be observed within the first 14 patients treated at the MTD, the trial was to be halted, because this event (0/14) has a probability of <0.05 if the true response rate is 0.20.

The study was conducted according to the ethical principles of the Declaration of Helsinki and Good Clinical Practice and was approved by health authorities and the independent ethics committee of the Academic Medical Center Amsterdam. The trial is registered on the USA NCI Web site www.ClinicalTrials.gov (NCT01077986)

### Patients

Patients with cytological or histological confirmed locally advanced or metastatic adenocarcinoma of the pancreas were eligible. Further inclusion criteria comprised an Eastern cooperative oncology group/World health organization (ECOG/WHO) performance status of 0, 1 or 2, measurable lesions on CT according to RECIST 1.0 criteria (for the phase II part of the study), age eighteen years of age or older and a life-expectancy of at least three months. Patients had to be mentally, physically and geographically able to undergo treatment and follow-up. Adequate renal, liver and bone marrow function was necessary. Laboratory values accompanied hereby were serum creatinine <150 μmol/L, bilirubin <1.5x upper limit of laboratory normal (ULN), aspartate aminotransferase and alanine aminotransferase <2.5x ULN or <5.0x in case of liver metastasis, white blood cell count >3.0x109, platelets >100x109, respectively.

Patients were not eligible if they had previous treatment with an mTOR inhibitor. Other exclusion criteria included pregnancy and lactation, clinical or radiological evidence of central nervous system metastasis at time of enrollment, known hypersensitivity to everolimus or other rapamycins or to its excipients, any severe and/or uncontrolled medical conditions, such as clinically significant heart conditions or myocardial infarction in 6 months prior to randomization, uncontrolled diabetes as defined by fasting glucose above 1.5x ULN, active or uncontrolled infection, serious liver disease and severely impaired lung function, or a serious concomitant systemic disorder that would compromise the safety of the patient, at the discretion of the investigator. Written informed consent was obtained from each patient.

### Treatment

Treatment was administered in a 3-week regimen consisting of continuous daily oral everolimus, weekly cetuximab, and capecitabine for 14 days followed by 7 days rest. For the phase I part, dose escalations were performed for everolimus and capecitabine, according to Table 1. Cetuximab was given at a fixed dose of 250 mg/m^2^, with a start-up dose of 400 mg/m^2^.

**Table 1:** Dose escalation levels phase I

Dose level	Everolimus (mg)	Capecitabine (bid mg/m^2^)	Cetuximab (mg)
−1	5	400	250
1	5	600	250
2	10	600	250
3	10	800	250

*bid* twice daily

If one of three patients experienced dose-limiting toxicity (DLT), three more patients were included at the same dose level. If two or more patients experienced DLT, the previous dose level was considered the MTD. All patients of the phase II part of the study were treated at the MTD.

DLTs were defined as any of the following adverse events as defined by the common terminology criteria for adverse events version 3.0 (CTCAE) in the first two cycles: grade 4 neutropenia lasting > 5 days or febrile neutropenia grade 3; grade 4 thrombocytopenia and grade ≥ 3 red cell count; grade ≥ 2 vomiting and grade ≥ 3 of any other toxicity despite supportive treatment, except rash, which was defined as DLT at grade 4.

In the phase II part, dose modifications were predefined for each drug. Everolimus dose was reduced in case of grade 3 toxicity or recurrence of grade 2 non-hematological toxicity or thrombocytopenia after interruption. Everolimus was discontinued in case of grade 4 toxicity or recurrence of grade 3 hematological toxicity after dose reduction. Capecitabine had to be withheld in case of toxicity grade ≥ 2 until recovery to grade ≤ 1. Dose modifications were dependent on severity and frequency of toxicity, as defined in the protocol. Cetuximab had to be delayed for up to two consecutive infusions in case of grade ≥3 skin toxicity whereas doxycyclin 100 mg daily and local metronidazole treatment was initiated. The same dose level was restarted if toxicity resolved to grade ≤ 2, with continuation of doxycyclin treatment. At second or third recurrence of grade 3 toxicity, dose was reduced to 200 mg/m^2^ and 150 mg/m^2^, respectively. Cetuximab was discontinued in case of withholding more than 2 infusions, fourth recurrence of skin toxicity grade ≥ 3, or an allergic/hypersensitivity reaction grade ≥ 3. Treatment was continued until unacceptable toxicity, disease progression, withdrawal of informed consent by the patient or any other reason why continuation was not in the best interest of the patient. Response assessment by CT-scan (RECIST 1.0) was done at baseline and every 9 weeks during active treatment.

## Results

### Patients

In total 43 patients were enrolled between February 2009 and June 2010. Three patients were excluded from analysis because of major violation of the inclusion criteria; one patient in the phase I part of the study received eight cycles of treatment, while in retrospect no pancreatic cancer cells were seen in pathology. Two patients in the phase II part experienced rapid deterioration between signing informed consent and start of treatment.

Table 2 summarizes the baseline characteristics of all 40 eligible patients, separately for each dose level.

**Table 2:** Patient demographics and disease characteristics

Characteristic	Phase II^a^	DL2
	N = 31 (%)	N = 9 (%)
Median Age	57.9	61
Range	39–78	45–69
Gender		
Male	13 (42)	6 (67)
Female	18 (58)	3 (33)
WHO performance status		
0	16 (52)	5 (56)
1	10 (32)	3 (33)
2	5 (16)	1 (11)
Stage of disease		
Locally advanced	4 (13)	1 (11)
Metastatic	27 (87)	8 (89)
Localization of primary		
Head	23 (74)	8 (89)
Tail/corpus	8 (26)	1 (11)
Line of therapy		
First line	22 (71)	6 (67)
≥ Second line	9 (29)	3 (33)

*WHO* World Health Organization *DL* Dose Level

^a^Phase II included 7 patients of the DL1 cohort and 24 patients of the phase II cohort

### Phase I

Sixteen patients were enrolled in the phase I part of this study. Dose level I was expanded to six patients because 1 patient developed grade 3 mucositis as DLT. The same patient discontinued treatment because of cerebral infarction 6 weeks after start of treatment. Because of vascular problems in the medical history of the patient, this complication was considered not to be related to study medication. Nonetheless, we decided to include an additional patient in this dose level. In the first cohort of dose level II one patient developed grade 3 hand-foot syndrome as DLT. Therefore this dose level was expanded to six patients. One of those patients developed grade 3 hand-foot syndrome and mucositis. These were not considered to be DLTs, because by mistake one additional week of capecitabine was taken by the patient in the first cycle and symptoms resolved after 3 weeks of interruption and the original dose could be restarted. Because two other patients with progressive disease within the first cycle, were not assessable for toxicity, three additional patients were included in DL2. Subsequently one patient developed grade 4 rash and three patients had grade 3 mucositis as DLTs. Due to the five DLTs in DL2, DL1 was considered as the MTD and the recommended dose for the phase II part.

In Table 4 all toxicity for the nine patients in dose level 2 is depicted. In these nine patients, a total of 25 complete cycles were given, with a median of 2 (range 0–10) cycles per patient. Dose reductions and interruptions are depicted in Table 3.

**Table 3:** Dose interruptions and reductions

	Interruptions		Reductions	
	Cycles (%)	Patients (%)	Cycles (%)	Patients (%)
Total DL2	10 (40)	5 (56)	5 (20)	5 (56)
Capecitabine	5 (20)	4 (44)	1 (4)	1 (11)
Everolimus	4 (16)	4 (44)	4 (16)	4 (44)
Cetuximab	1 (4)	1 (11)	0 (0)	0 (0)
Total phase 2	22 (20)	8 (26)	6 (5)	6 (19)
Capecitabine	6 (5)	3 (10)	5 (4)	5 (16)
Everolimus	5 (4)	5 (16)	1 (1)	1 (3)
Cetuximab	11 (10)	5 (16)	0 (0)	0 (0)

Total cycles: 25 in dose level 2 (DL2), 113 in phase 2. Total patients: 9 in DL2 and 31 in phase 2

### Phase II

#### Safety and toxicity

In the phase II part of this study, 24 eligible patients were enrolled. When combined with the seven patients treated at MTD in the phase I part, a total of 31 patients could be evaluated according to the protocol. In these 31 patients, a total of 113 complete cycles were given, with a median (range) of 3.0 (0–20) cycles per patient. Dose reductions and interruptions are depicted in Table 3. Table 4 represents the treatment related toxicity. The most common grade 3/4 toxicities of the phase II part included hyperglycemia (26%), rash (19%), mucositis (13%) and fatigue (13%).

**Table 4:** Treatment related toxicity

	31 patients in phase II analysis^a^		9 patients in DL2	
	Any grade (%)	Grade 3–4 (%)	Any grade (%)	Grade 3–4 (%)
Hematologic				
Anemia	14 (45)	1 (3)	6 (67)	0 (0)
Neutropenia	7 (23)	0 (0)	5 (56)	0 (0)
Thrombocytopenia	8 (26)	0 (0)	3 (33)	0 (0)
Non hematological				
Rash	24 (77)	6 (19)	6 (67)	3 (27)
Mucositis	14 (45)	4 (13)	6 (67)	4 (36)
Fatigue	13 (42)	4 (13)	4 (44)	1 (9)
Diarrhea	10 (32)	4 (13)	3 (33)	0 (0)
Hand-foot syndrome	10 (32)	2 (6)	1 (11)	2 (18)
Infection	5 (16)	1 (3)	2 (22)	2 (18)
Nausea/vomit	16 (52)	1 (3)	6 (67)	0 (0)
Hypokalemia	17 (55)	4 (13)	1 (11)	0 (0)
Hyperglycaemia	19 (61)	8 (26)	5 (56)	1 (18)
Total unique patients	31 (100)	23 (74)	9 (100)	8 (89)

*DL* Dose Level ^a^ Phase II includes 7 patients from DL1 and 24 from phase II

#### Efficacy and survival

Table 5 summarizes the objective response observed in the 31 treated patients (71% first line, 29% second line) at the MTD level. Out of 31 patients, 24 patients were evaluable for response. Seven patients were not evaluable for response due to events before the first planned CT-evaluation: two patients were clinically progressive, one patient died (as described in the phase I part) and three patients stopped treatment because of treatment related toxicity (grade 3 diarrhea, intolerable rash). One patient refused further treatment. These seven patients were considered as non-responders.

**Table 5:** Objective treatment response

Response	*N* = 31(%)	CI
PR	2 (6.5)	1.1–18.8
SD	5 (16.1)	6.2–31.2
PD	17 (54.8)	37.9–71.1
Not assessed	7 (22.6)	10.6–38.8
Objective Response rate^a^	2 (6.5)	1.1–18.8
Disease Control rate^b^	7 (22.6)	10.6–38.8

*PR* partial response, *SD* stable disease *PD* progressive disease, *CI* confidence interval ^a^ Objective response rate = PR ^b^ Disease Control rate = PR + SD

We observed two (6.5% CI: 1.1–18.8%) partial responses according to RECIST 1.1. Five (16.1% CI: 6.2–31.2%) patients had stable disease, giving a disease control rate of 23.8%. Seventeen (54.8% CI: 37.9–71.1%) patients had progressive disease.

**Fig. 1:**
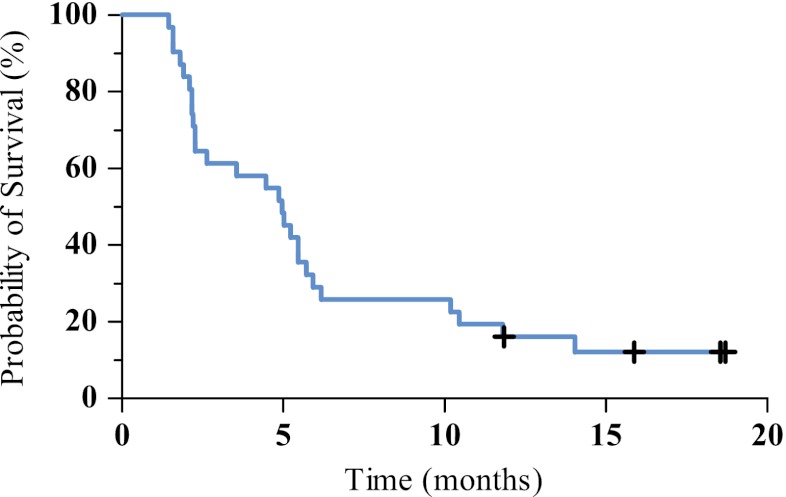
Kaplan Meier curve of overall survival of all patients treated at the maximum tolerated dose. Median survival is 5.0 months (CI 3.1–6.8) + *censored patients*

Overall survival of the 31 patients treated at MTD was 5.0 months (CI 3.1–6.8 months) (Fig. 1). The one-year survival rate was 12,9%. Time to treatment failure was 2.3 months (CI 1.7–2.8 months).

Table 6 summarizes overall survival differences between groups based on differences in baseline characteristics, measured by KM-method and a log-rank test. Overall survival for first line patients was 5.0 (CI 4.1–5.9) months, and for second line patients 3.6 months (CI 0.0–7.7) months, which was not a significant difference.

**Table 6:** Kaplan-Meyer analysis for overall survival, effect of baseline characteristics

	Total	Censored	Median survival (Months, CI)	1 year survival (%)	Logrank_p
All Patients	31	4	5.0 (3.1–6.8)	12.9	
Sex					
Female	18	0	5.0 (4.2–5.7)	5.6	0.203
Male	13	4	4.5 (1.7–7.3)	23.1	
Age					
<65	24	2	4.5 (1.2–7.7)	12.5	0.112
≥65	7	2	10.2 (0.0–20.5)	14.3	
WHO performance status					
0	16	4	5.2 (4.1–6.4)	25.0	0.093
1	10	0	4.5 (0.1–8.8)	0.0	
2	5	0	2.3 (1.9–2.7)	0.0	
Metastasis					
Locally advanced	4	1	5.2 (2.0–8.4)	25.0	0.547
Metastatic	27	3	4.9 (2.5–7.3)	11.1	
Line of therapy					
First line	22	3	5.0 (4.1–5.9)	18.2	0.603
≥Second line	9	1	3.6 (0.0–7.3)	0.0	

*WHO* World Health Organization *CI* Confidence Interval

## Discussion

The MTD for everolimus and capecitabine in combination with cetuximab (cetuximab 250 mg/m^2^ weekly) was already reached at the first dose level (everolimus 5 mg daily, and capecitabine 600 mg/m^2^ BID). The DLTs were mucositis, rash and hand-foot syndrome. In the phase II part of this study the incidence of grade 3–4 hyperglycemia, a well known complication of everolimus, was considerable and seems even to be higher compared to studies with everolimus alone. Despite the relative low dose level of everolimus (5 mg) the incidence of severe mucositis (13% grade 3–4) was still considerable and also seems to be higher compared to trials with everolimus monotherapy (10 mg). In other studies using single agent everolimus the incidence of grade 3–4 mucositis was 1–7% [26, 27]. In our previous phase I study (Deenen et al.) with everolimus 10 mg and capecitabine 500–1000 mg/m^2^, mucositis was not dose-limiting, however grade 1–2 mucositis was present in 50% of the patients [21]. Thus, the addition of cetuximab resulted in more toxicity and prevented dose escalations of everolimus and capecitabine to more optimal dosages [28]. Although the underlying mechanism is unclear, co- administration of three agents with overlapping toxicities may be an important explanation for the excessive mucosal and/or epidermal toxicities seen in this study. Because the study of Deenen et al. demonstrated no pharmacokinetic interactions between everolimus and capecitabine, and monoclonal antibodies do not interfere at pharmacokinetic level, the increased toxicity is probably caused by pharmacodynamic interaction between the three drugs.

The objective response rate was only 6.5% (CI 1.1–18.8) with an overall survival of 5 months. In the first line cohort the OS was also 5.0 months, which even seems to be slightly inferior in comparison with gemcitabine as first line treatment [2]. In preclinical studies with cell lines of non small cell lung, pancreatic, colon, and breast cancer combined inhibition of mTOR and EGFR resulted in a potentiation of anti cancer activity and resensitization of cell lines resistant to EGFR inhibitors [15–18, 29]. Despite these promising preclinical results, exploration of this strategy in pancreatic cancer patients in the present study was disappointing. Possible reasons for clinical resistance to this treatment combination are the following. Toxicity prevented the administration of everolimus at optimal dosages. Everolimus shows most effective mTOR inhibition at a dose of 10 mg, and capecitabine monotherapy showed the optimal efficacy at 1000–1250 mg/m^2^ (BID) [28, 30, 31]. Secondly, the desmoplastic nature of pancreatic cancer with a high fibrotic component and minimal vascularization might prevent an adequate drug penetration, especially a large molecule like the monoclonal antibody cetuximab. Another, in various cancer types well established, cause of resistance to anti-EGFR treatment is KRAS mutation, predominantly present in pancreatic cancer cells, which accounts for constitutive signaling directly downstream of EGFR. However, inhibitors of EGFR still show efficacy in some KRAS mutated pancreatic cancer cell lines [32].

In conclusion, dose escalation of everolimus and capecitabine in the present triple drug schedule with cetuximab was not possible because of severe, especially epidermal and mucosal, toxicity. At the relative low everolimus and capecitabine dosages in the phase II part of this study the toxicity was still considerable, leading to dose interruptions and adaptations. Considering the toxicity, the response rate of 6.5%, and median survival of 5 months in first line treated patients, this regimen does not deserve further exploration in pancreatic cancer patients.
